# Real-Time Analytics and AI for Managing No-Show Appointments in Primary Health Care in the United Arab Emirates: Before-and-After Study

**DOI:** 10.2196/64936

**Published:** 2025-01-06

**Authors:** Yousif Mohamed AlSerkal, Naseem Mohamed Ibrahim, Aisha Suhail Alsereidi, Mubaraka Ibrahim, Sudheer Kurakula, Sadaf Ahsan Naqvi, Yasir Khan, Neema Preman Oottumadathil

**Affiliations:** 1 Emirates Health Services, Dubai, United Arab Emirates; 2 Oracle, The Edge Building, Al Falak Street, Dubai Internet City, Dubai, United Arab Emirates, 971 558620820

**Keywords:** electronic health record, EHR, artificial intelligence, AI, no-show appointments, real-time data, primary health care, risk prediction, clinic waiting time, operational efficiency

## Abstract

**Background:**

Primary health care (PHC) services face operational challenges due to high patient volumes, leading to complex management needs. Patients access services through booked appointments and walk-in visits, with walk-in visits often facing longer waiting times. No-show appointments are significant contributors to inefficiency in PHC operations, which can lead to an estimated 3%-14% revenue loss, disrupt resource allocation, and negatively impact health care quality. Emirates Health Services (EHS) PHC centers handle over 140,000 visits monthly. Baseline data indicate a 21% no-show rate and an average patient wait time exceeding 16 minutes, necessitating an advanced scheduling and resource management system to enhance patient experiences and operational efficiency.

**Objective:**

The objective of this study was to evaluate the impact of an artificial intelligence (AI)-driven solution that was integrated with an interactive real-time data dashboard on reducing no-show appointments and improving patient waiting times at the EHS PHCs.

**Methods:**

This study introduced an innovative AI-based data application to enhance PHC efficiency. Leveraging our electronic health record system, we deployed an AI model with an 86% accuracy rate to predict no-shows by analyzing historical data and categorizing appointments based on no-show risk. The model was integrated with a real-time dashboard to monitor patient journeys and wait times. Clinic coordinators used the dashboard to proactively manage high-risk appointments and optimize resource allocation. The intervention was assessed through a before-and-after comparison of PHC appointment dynamics and wait times, analyzing data from 135,393 appointments (67,429 before implementation and 67,964 after implementation).

**Results:**

Implementation of the AI-powered no-show prediction model resulted in a significant 50.7% reduction in no-show rates (*P*<.001). The odds ratio for no-shows after implementation was 0.43 (95% CI 0.42-0.45; *P*<.001), indicating a 57% reduction in the likelihood of no-shows. Additionally, patient wait times decreased by an average of 5.7 minutes overall (*P*<.001), with some PHCs achieving up to a 50% reduction in wait times.

**Conclusions:**

This project demonstrates that integrating AI with a data analytics platform and an electronic health record systems can significantly improve operational efficiency and patient satisfaction in PHC settings. The AI model enabled daily assessments of wait times and allowed for real-time adjustments, such as reallocating patients to different clinicians, thus reducing wait times and optimizing resource use. These findings illustrate the transformative potential of AI and real-time data analytics in health care delivery.

## Introduction

Primary health care (PHC) centers are the initial contact point for health care and are considered the gateway to the health care system for a large proportion of the population [1,2]. PHC centers experience a high daily patient influx, making PHC operations complex and requiring efficient systems. While many patients book appointments in advance, others rely on walk-ins, often leading to longer wait times for older and pediatric patients who require quicker attention. However, a significant issue is no-show appointments, where patients fail to attend their scheduled appointments without prior cancellation.

No-show appointments create a significant burden by unnecessarily blocking health care resources. They can negatively impact the financial and operational planning of health care organizations, leading to an estimated revenue loss of 3%‐14% [3,4]. Various interventions have been proposed to address this issue, focusing on understanding patient behaviors and reducing no-show rates [5]. Many organizations use data analytics to predict no-show probabilities and devise interventions to improve clinical efficiencies [6-8]. Literature suggests that while machine learning models have been explored to predict no-show likelihoods [9], a gap exists in operationalizing these insights at a large scale, especially in settings like the Emirates Health Services (EHS), which manages a vast network of health care facilities.

Another key indicator of patient satisfaction is waiting time, which directly affects the quality of care and patient experience [10-12]. Numerous studies have demonstrated that reducing patient waiting times can significantly enhance patient satisfaction and overall service quality. These studies highlight that multiple interventions, such as resource optimization and system redesign, are necessary to improve operational efficiency. Identifying bottlenecks and implementing practical interventions can significantly reduce waiting times [13-17]. With the evolution of artificial intelligence (AI), organizations can now leverage real-time data analytics to develop predictive models and suggest proactive interventions.

While there are examples from various health care settings demonstrating the successful use of AI models and patient reminders to reduce no-shows [18,19], there is limited research on how such systems can be optimized specifically for large health care networks like the EHS. Several studies highlight the importance of real-time analytics in improving health care outcomes, allowing organizations to respond promptly to emerging challenges and optimize resource allocation [20-22]. Hence, this study aims to address this gap by providing an electronic health record (EHR)-driven, real-time analytics solution tailored to the operational needs of PHC centers.

The EHS is a vast health care network across 6 emirates in the United Arab Emirates, managing hospitals, clinics, specialized centers, and supporting services [23]. The PHC centers within the EHS handle over 140,000 visits each month, resulting in a high patient turnover. While this often leads to longer-than-expected wait times negatively impacting patient experience, some appointment slots remain underused due to an average no-shows rate of 21%. Baseline data show the average physician waiting time exceeds 16 minutes, and while resources are distributed based on center needs, occasional shortages contribute to operational delays.

Considering these challenges, our study aims to evaluate the impact of a real-time dashboard that projects operational metrics and integrates an AI model to predict no-show appointments in PHC centers at the EHS. By using this AI-enriched dashboard, clinic administrators can identify high-risk patients (risk of no-show) and proactively contact them to emphasize the importance of their scheduled visits. The primary goals of this evaluation were to assess if the dashboard improved operational efficiency, reduced no-show appointments, optimized resource allocation, and decreased patient waiting times.

## Methods

### Study Design

This project was conceived as an innovative data-driven intervention using our data intelligence platform seamlessly integrated with the EHR (Multimedia Appendix 1). Using real-time PHC data, we created a dashboard with an AI model to guide the operational workflow and to reduce clinic wait times and no-show appointments.

This was a before-and-after study that evaluated the impact of a real-time dashboard with an AI-powered no-show prediction model at EHS PHCs 3 months before and after implementation. By leveraging this dashboard, clinic coordinators could proactively manage PHC traffic flow, enhancing operational efficiency and reducing patient waiting times. To capture key metrics accurately, several data definitions were established to track patient flow within the PHC setting. Clinic waiting time was defined as the intervals between various points in a patient’s journey, which were all recorded as time stamps in the EHR system. Specifically, this included:

- Nurse waiting time: time from registration to the nurse station.
- Physician waiting time: time from being ready to be seen by the physician to the actual consultation.
- Check-in to checkout time: total time from check-in to final checkout by the physician.
- Appointment no-show: defined as a patient missing a scheduled appointment without prior notice to the PHC.

For comprehensive analysis, all visit and attendance data were sourced from the EHR and the human resources attendance system, both of which were updated in real time to ensure data accuracy and relevance.

### Research Road Map Intervention and Dashboard Implementation

Our research followed a structured path from data acquisition to intervention and evaluation. We first integrated patient and clinic staff data from the EHR into a real-time dashboard that incorporated an AI model trained on historical EHR data to predict no-show appointments. This model, along with other key PHC metrics—such as footfall, resource availability, and wait times—were displayed on the dashboard to support clinic coordinators in managing appointments proactively. The AI model projected daily no-show risk, allowing coordinators to reach out to high-risk patients for confirmation or rescheduling. Additionally, real-time resource constraints were visible on the dashboard, enabling operational adjustments as needed. All users received training on the new dashboard and workflow, and continuous monitoring and evaluation processes were established to assess ongoing performance. Finally, we analyzed the results to measure the dashboard’s impact on clinic operations (MultimediaAppendices 2  3).

### AI Model

Our AI model was developed to predict no-show appointments using historical EHR data from EHS PHCs, incorporating 16 distinct features: 4 demographic, 3 on patient history, and 9 appointment-specific factors (Multimedia Appendix 4). We used a random forest classification technique to assess the model’s effectiveness in handling high-dimensional data, achieving an accuracy rate of 86%. The model categorized appointments into a high, medium, or low risk of a no-show, based on probability thresholds of ≥90%, 89%‐80%, and 79%‐70%, respectively. To validate the AI model, we used bootstrapping to ensure robustness and to reduce overfitting.

### Statistical Analysis and Experimental Validation

Data were analyzed using SPSS version 17 (IBM Corp) and SAS version 03.05 software (SAS Institute). Descriptive statistics were computed for all variables. Independent 2-tailed sample *t* tests were conducted to compare mean waiting times before and after the intervention. To analyze changes in appointment no-show rates, *z* tests were performed. Logistic regression was used to estimate odds ratios (OR) and determine the likelihood of a no-show after implementation. Statistical significance was set at an α level of .05, with 95% CI computed for all estimates. The statistical validation involved performing hypothesis tests to determine the significance of changes in waiting times and no-show rates.

### Outcome Measures

The outcome measures were the mean difference in waiting times, which was calculated using the waiting times before and after implementation and the percent reduction in no-shows, which was calculated using the no-show rates before and after implementation.

### Ethical Considerations

Ethical approval was obtained from the Dubai Research Ethics Committee at the Ministry of Health and Prevention, United Arab Emirates. (Approval Reference No. MOHAP/DXB-REC/S.O.O/No.136/2023). The study involved deidentified, retrospective data negating the need for individual patient consent. Patient compensation was not applicable for this study. Bias was minimized by using deidentified data and implementing objective data quality control measures.

## Results

We implemented our project across all PHCs in the EHS in October 2022. To compare the outcomes of this tool, we analyzed the data 3 months before (July to September 2022) and 3 months after (November 2022, to January 2023) implementation, and the outcomes were grouped and labeled as before or after, respectively. Descriptive analytics revealed a total of 67,429 booked visits before implementation compared to 67,964 visits after implementation with a similar sex and nationality distribution between both periods (Multimedia Appendix 5). Our analysis showed that most of the visits were through walk-ins instead of booked appointments. There was a slight decrease in walk-in appointments between the two periods, but the percentage of these visits remained relatively stable (80.9% to 80.7%). There was a notable change in the number of no-show appointments, drastically reducing from 20.82% to 10.25% (Table 1).

**Table 1.** Visit frequencies and dynamics before and after program implementation in primary health care.

	Before implementation, n/N (%)	After implementation, n/N (%)
Walk-ins	285,700/353,129 (80.91)	284,015/351,979 (80.69)
Appointments	67,429/353,129 (19.09)	67,964/351,979 (19.31)
No-shows	14,038/67,429 (20.82)	6966/67,964 (10.25)

Using a *z* test, our analysis showed that the implementation of the no-show AI model led to a significant 50.7% reduction in no-show rates across the PHC department (*P*<.001). Binary logistic regression further assessed the dashboard’s impact on reducing no-shows, yielding an OR of 0.43 (95% CI 0.42‐0.45; *P*<.001). This OR being less than 1 indicated that the intervention effectively decreased no-show occurrences, reducing the likelihood of no-shows by 57% after implementation of the AI model (Table 2).

**Table 2.** Mean difference and impact analysis of appointment no-show rates before and after program implementation in primary health care centers.

Measurements	Values	*P* value
Difference in no-show rate after implementation, n/N (%)	50.7	<.001
Likelihood of no-shows after implementation, odds ratio (95% CI)	0.43 (0.42-0.45)	<.001

Table 3 reflects the different waiting times for the nurse assessment, physician assessment, and overall consultation time (Multimedia Appendix 6). We observed reduced average waiting times at all stations across the PHC journey. We found a decrease in patient wait times by an average of 5.7 minutes (from 54 to 49 minutes; *P*<.001). Based on the average time saved per visit (5.7 minutes), a total of 387,394.8 minutes were saved in the 3 months after implementation. This is equivalent to approximately 6,456.6 hours saved during this period. The reduction in waiting time from check-in to checkout has been consistent since implementing this solution.

**Table 3.** Comparison of patient waiting times at different stations during patient visits before and after program implementation at primary health care centers.

Waiting times	Before implementation (min), mean (SE)	After implementation (min), mean (SE)	Difference following implementation (min)	*P* value
Nurse waiting time	8.4 (0.02)	7.2 (0.02)	1.2	<.001
Physician waiting time	16.3 (0.03)	15.0 (0.03)	1.3	<.001
Physician consultation to checkout time	29.3 (0.06)	26.2 (0.06)	3.1	<.001
Check-in to checkout time	54.2 (0.08)	48.5 (0.07)	5.7	<.001

## Discussion

### Principal Results and Comparison With Prior Work

The implementation of an AI-powered no-show prediction model, integrated with a real-time operational dashboard at PHC centers within the EHS, has demonstrated significant improvements in operational efficiency and patient care. Our main findings revealed a significant 50.7% reduction in no-show appointments *(P*<.001), and a 5.7-minute reduction in overall patient waiting times, highlighting the direct impact of our intervention on streamlining health care delivery. These results underlined the value of leveraging predictive analytics and real-time data in optimizing resource allocation, reducing manual processes, and enhancing the patient experience. Unlike other studies, where the emphasis has been to explore the factors and behaviors contributing to patient no-shows using machine learning and other techniques [24], we successfully operationalized our machine learning model and connected it to a closed loop workflow which helped reduce absenteeism.

A key achievement of this intervention was the substantial reduction in no-show appointments. By proactively contacting high-risk patients identified by the AI model, clinic administrators were able to significantly reduce missed appointments. This was a notable success, as it not only improved the efficiency of the appointments but also allowed walk-in patients to fill the available slots, further maximizing clinic capacity. Additionally, the use of real-time dashboards provided clinic managers with immediate insights into patient volumes, waiting times, and staff availability, enabling swift adjustments in resource allocation during peak hours. These operational efficiencies, particularly the reduction in waiting times at multiple stages of the patient journey (nurse assessment, physician consultation, and overall clinic visit), underscore the importance of real-time analytics in improving the flow of care. Our robust analysis reassured that the findings of this before and after implementation were significant and could be attributed to our intervention.

The impact of these factors has been previously studied in isolation. There are successful examples of AI models to predict no-show appointments, which have helped reduce the burden of these instances [25-27]. Our observations were in alignment with these studies. Like other studies, we have seen that connecting with patients before their scheduled appointment reduces their chances of missing it [28]. Several strategies, like text messages, patient portal reminders, and phone calls, have been examined, and it has been found that calling patients before their scheduled visit is effective for reducing no-shows [29].

Operationally, real-time dashboards and decision support tools for clinic managers are helpful for identifying operational bottlenecks. Numerous studies have examined the impact of real-time dashboards on reducing patient waiting times in health care settings. These studies suggest that real-time dashboards are crucial for improving patient flow and reducing waiting times across various health care settings, emphasizing their potential as valuable tools in optimizing health care delivery [30-33].

As recommended by other studies, using data to calculate and predict interday scheduling precisely is undeniably beneficial for large health care organizations [30,34]. Trend analysis helps estimate the expected number of unbooked versus booked visits and helps allocate resources accordingly. Many PHC centers reserve a proportion of their scheduling slots for walk-in visits. Since the EHS has many walk-in visits, we should follow a similar strategy and use predictive analysis to help improve our resource allocation. We must carefully design this strategy to reduce our patient waiting times. In the future, we could further enhance the outcomes achieved in this study. By scaling up the AI-model to other services and departments, we could increase the benefits of this program for the EHS. The flexibility and the scalability of our real-time data intelligence platform makes it a valuable tool to resolve operational challenges in our health care system.

### Strengths

This study presents a novel approach by integrating an AI-driven predictive model with a real-time dashboard to manage no-show appointments and reduce waiting times in a PHC setting. Unlike previous studies, which primarily focus on predictive modeling without an operational framework, our approach goes a step further by embedding predictive insights into an actionable, real-time dashboard that enables proactive intervention. By operationalizing predictive analytics within a large-scale health care network, this study contributes a scalable and practical solution to the current state of digital health interventions aimed at improving health care efficiency and patient outcomes.

The major strengths of our study include big data analysis, the use of case mix, the contextualization to the local population, crossfunctional analytics for the clinician-to-patient ratio, real-time analysis for immediate identification of prolonged waiting times, and AI-based modeling for no-show appointment prediction that is coupled with patient contact information. Our holistic approach to use an AI model and real-time insights for a guided operational management workflow is unique in its nature and it can be used by organizations with similar resources.

### Limitations

Certain limitations of this study must be acknowledged. First, the mitigation workflow is outside of EHRs, so we cannot quantify the efforts accurately. It is an established fact that a reduction in no-show appointments result in improved financial outcomes [35,36], and we acknowledge that a detailed analysis of the financial implications of reducing no-shows was beyond the scope of this study. Future research could address this aspect to provide a more comprehensive understanding of the intervention’s economic impact.

### Conclusion

In conclusion, this project represents the first instance of the EHS using a real-time, AI-driven analytics platform to improve operational efficiency, reduce no-show appointments, and enhance patient experience. The success of our solution in reducing no-shows and waiting times highlights the importance of integrating advanced analytics into daily health care operations. By providing clinic administrators with real-time actionable data, this platform enables better decision-making, optimized resource use, and improved patient flow. The scalability of this application across other health care settings further emphasizes its potential to drive widespread improvements in health care delivery, making it a valuable tool for health care organizations looking to modernize their operations through data-driven solutions. As we continue to refine this system, we expect further reductions in waiting times and improvements in patient satisfaction.

## Supplementary material

10.2196/64936 Multimedia Appendix 1 Data architecture diagram representing the layers of data transition from source of information to the dashboard.

10.2196/64936 Multimedia Appendix 2 Program workflow for using the real-time artificial intelligence–powered dashboard to manage no-shows and reduce waiting times in primary health care.

10.2196/64936 Multimedia Appendix 3 A screenshot of the data dashboard showing real-time analytics for primary health care visits in a day.

10.2196/64936 Multimedia Appendix 4 Major predictors contributing to the no-show prediction artificial intelligence model from primary health care visit data.

10.2196/64936 Multimedia Appendix 5 Frequency distribution of booked visits before and after the artificial intelligence program implementation in primary health care centers (July 2022, to January 2023).

10.2196/64936 Multimedia Appendix 6 Reduction in patient waiting times at different stages of care following artificial intelligence program implementation in primary health care centers.

## Abbreviations

AI: artificial intelligence
EHR: electronic health record
EHS: Emirates Health Services
OR: odds ratio
PHC: primary health care
