# Supplementary material for: Real-Time Analytics and AI for Managing No-Show Appointments in Primary Health Care in the United Arab Emirates: Before-and-After Study
Source: JMIR Form Res. 2025 Jan 6;9:e64936. doi: 10.2196/64936 (PMC11729783; doi:10.2196/64936)
Supplement: Multimedia Appendix 1 [file formative-v9-e64936-s001.pptx]

## Slide 1
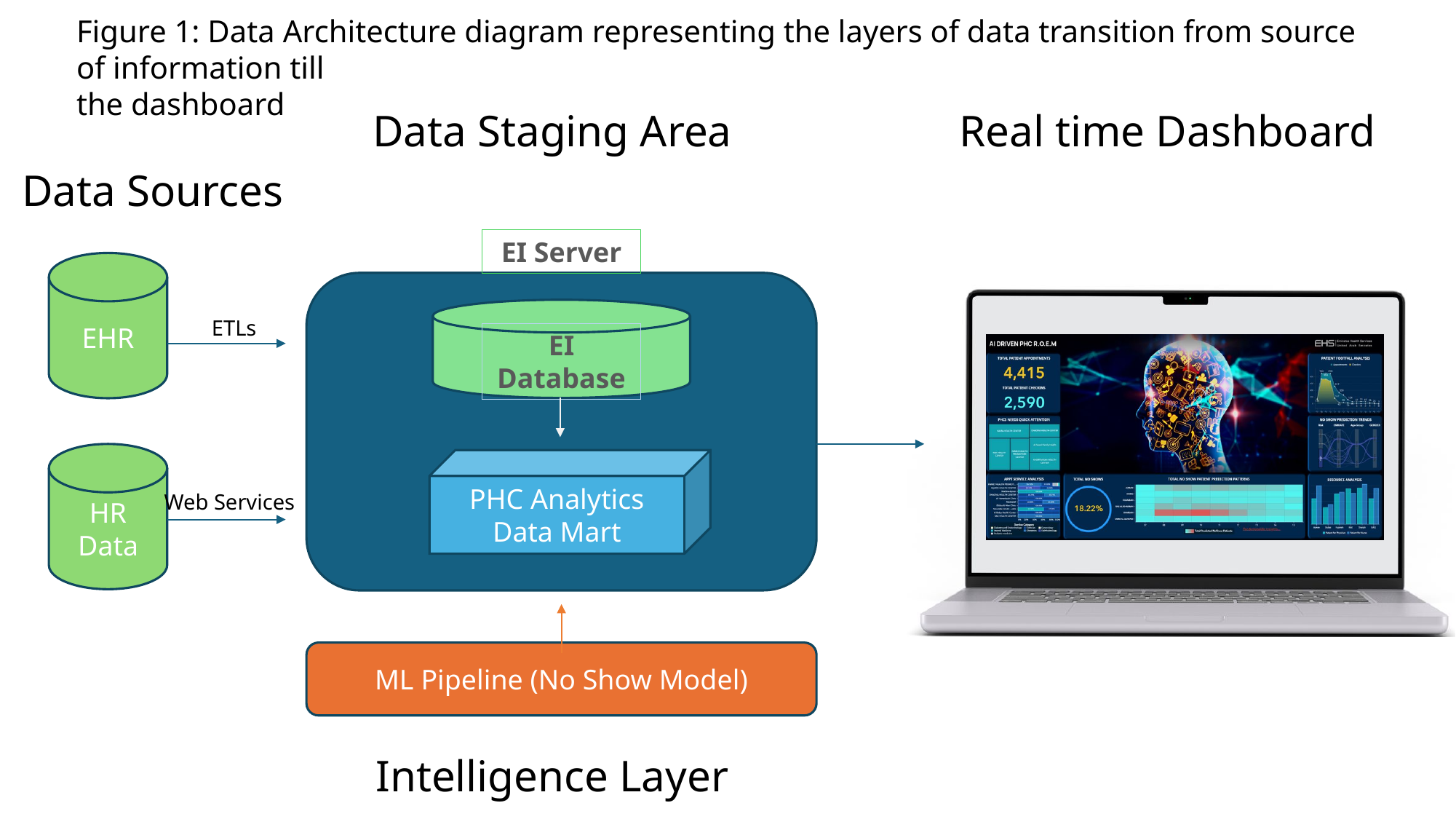

Figure 1: Data Architecture diagram representing the layers of data transition from source of information till
the dashboard
Data Staging Area
Real time Dashboard
Data Sources
EI Server
EHR
ETLs
EI Database
HR Data
PHC Analytics Data Mart
Web Services
ML Pipeline (No Show Model)
Intelligence Layer
