# Supplementary material for: Real-Time Analytics and AI for Managing No-Show Appointments in Primary Health Care in the United Arab Emirates: Before-and-After Study
Source: JMIR Form Res. 2025 Jan 6;9:e64936. doi: 10.2196/64936 (PMC11729783; doi:10.2196/64936)
Supplement: Multimedia Appendix 2 [file formative-v9-e64936-s002.pptx]

## Slide 1
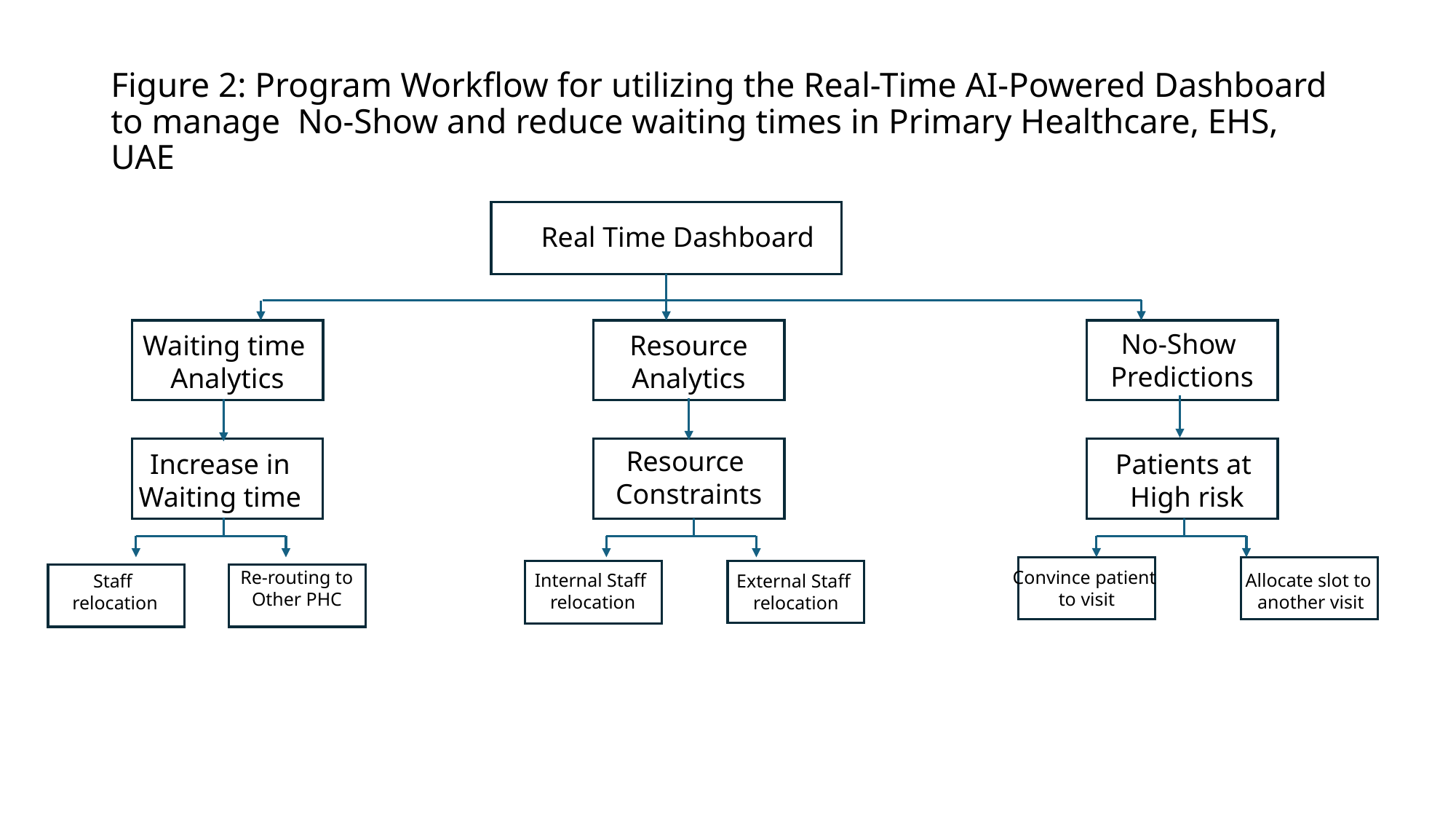

# Figure 2: Program Workflow for utilizing the Real-Time AI-Powered Dashboard to manage No-Show and reduce waiting times in Primary Healthcare, EHS, UAE
Real Time Dashboard
No-Show
Predictions
Waiting time
Analytics
Resource
Analytics
Resource
Constraints
Increase in
Waiting time
Patients at
High risk
Re-routing to
Other PHC
Convince patient
to visit
Allocate slot to
another visit
Internal Staff
relocation
External Staff
relocation
Staff
relocation
