# Supplementary material for: Real-Time Analytics and AI for Managing No-Show Appointments in Primary Health Care in the United Arab Emirates: Before-and-After Study
Source: JMIR Form Res. 2025 Jan 6;9:e64936. doi: 10.2196/64936 (PMC11729783; doi:10.2196/64936)
Supplement: Multimedia Appendix 3 [file formative-v9-e64936-s003.pptx]

## Slide 1
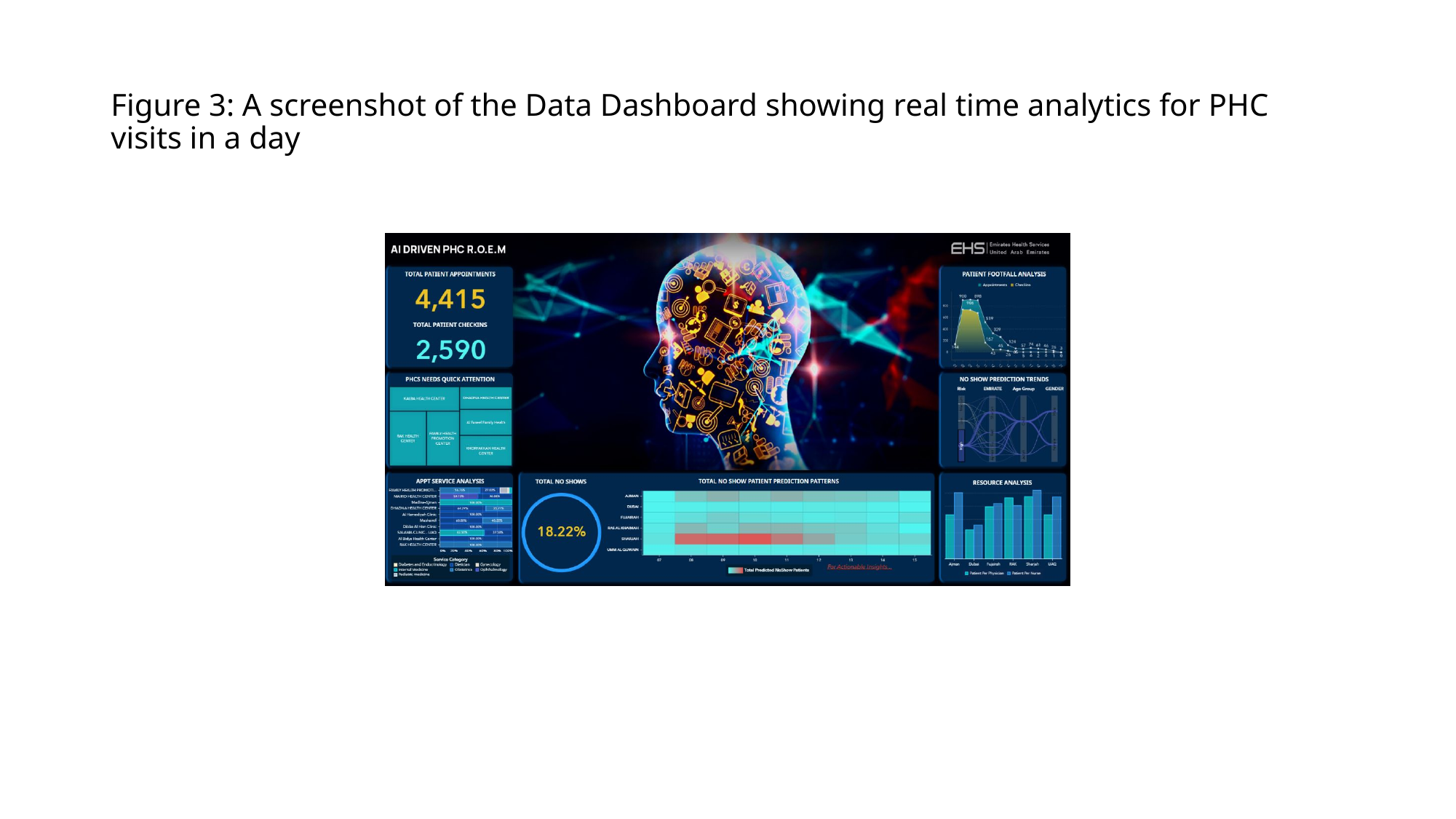

# Figure 3: A screenshot of the Data Dashboard showing real time analytics for PHC visits in a day
