# Supplementary material for: Real-Time Analytics and AI for Managing No-Show Appointments in Primary Health Care in the United Arab Emirates: Before-and-After Study
Source: JMIR Form Res. 2025 Jan 6;9:e64936. doi: 10.2196/64936 (PMC11729783; doi:10.2196/64936)
Supplement: Multimedia Appendix 4 [file formative-v9-e64936-s004.pptx]

## Slide 1
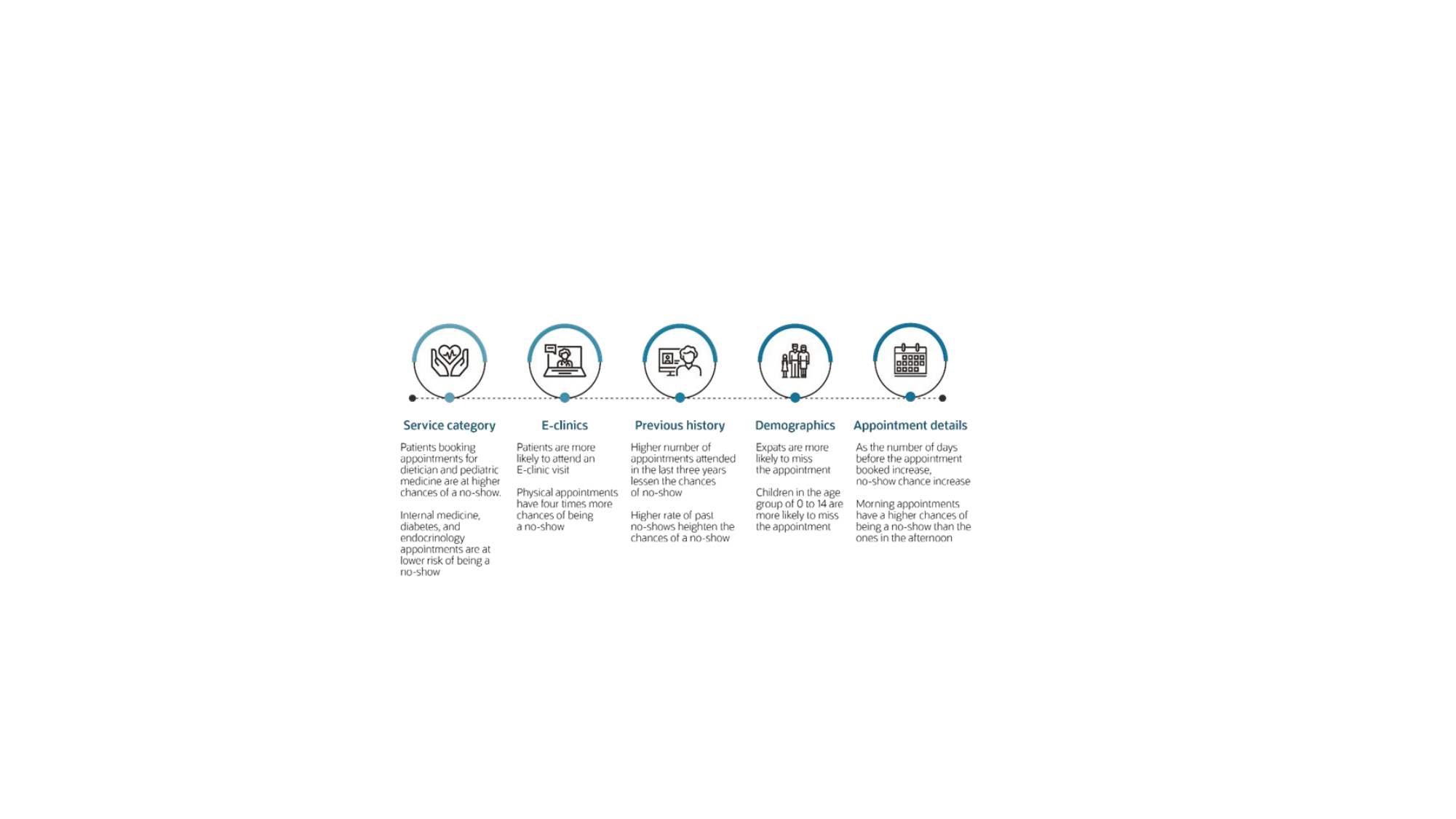

## Slide 2
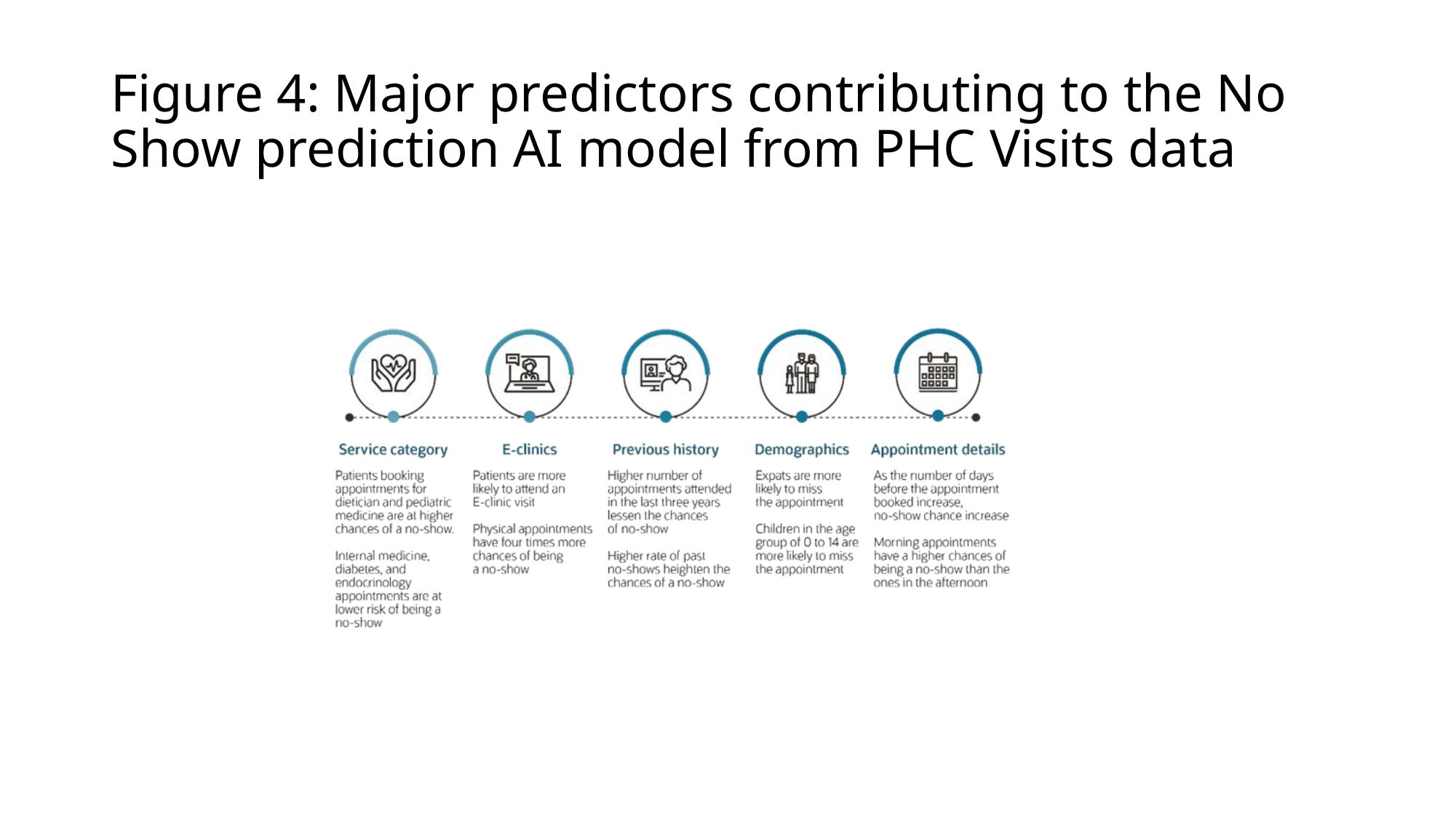

# Figure 4: Major predictors contributing to the No Show prediction AI model from PHC Visits data
