# Supplementary material for: Real-Time Analytics and AI for Managing No-Show Appointments in Primary Health Care in the United Arab Emirates: Before-and-After Study
Source: JMIR Form Res. 2025 Jan 6;9:e64936. doi: 10.2196/64936 (PMC11729783; doi:10.2196/64936)
Supplement: Multimedia Appendix 5 [file formative-v9-e64936-s005.pdf]

	Before (Jul-Sep’22)	Percentage	After (Nov’22-Jan’23)	Percentage
Total outpatient appointments (Excluded walk-ins)	67,429	100	67,964	100
Males	27,116	40.2	27,870	41.0
Females	40,311	59.8	40,091	59.0
Emiratis	48,595	72.1	49,451	72.8
Expats	18,833	27.9	18,513	27.2

Table 1- Frequency Distribution of Booked Visits Pre- and Post-AI Program Implementation in Primary Healthcare Centres, UAE (July 2022 - January 2023)
