# Supplementary material for: Real-Time Analytics and AI for Managing No-Show Appointments in Primary Health Care in the United Arab Emirates: Before-and-After Study
Source: JMIR Form Res. 2025 Jan 6;9:e64936. doi: 10.2196/64936 (PMC11729783; doi:10.2196/64936)
Supplement: Multimedia Appendix 6 [file formative-v9-e64936-s006.pptx]

## Slide 1
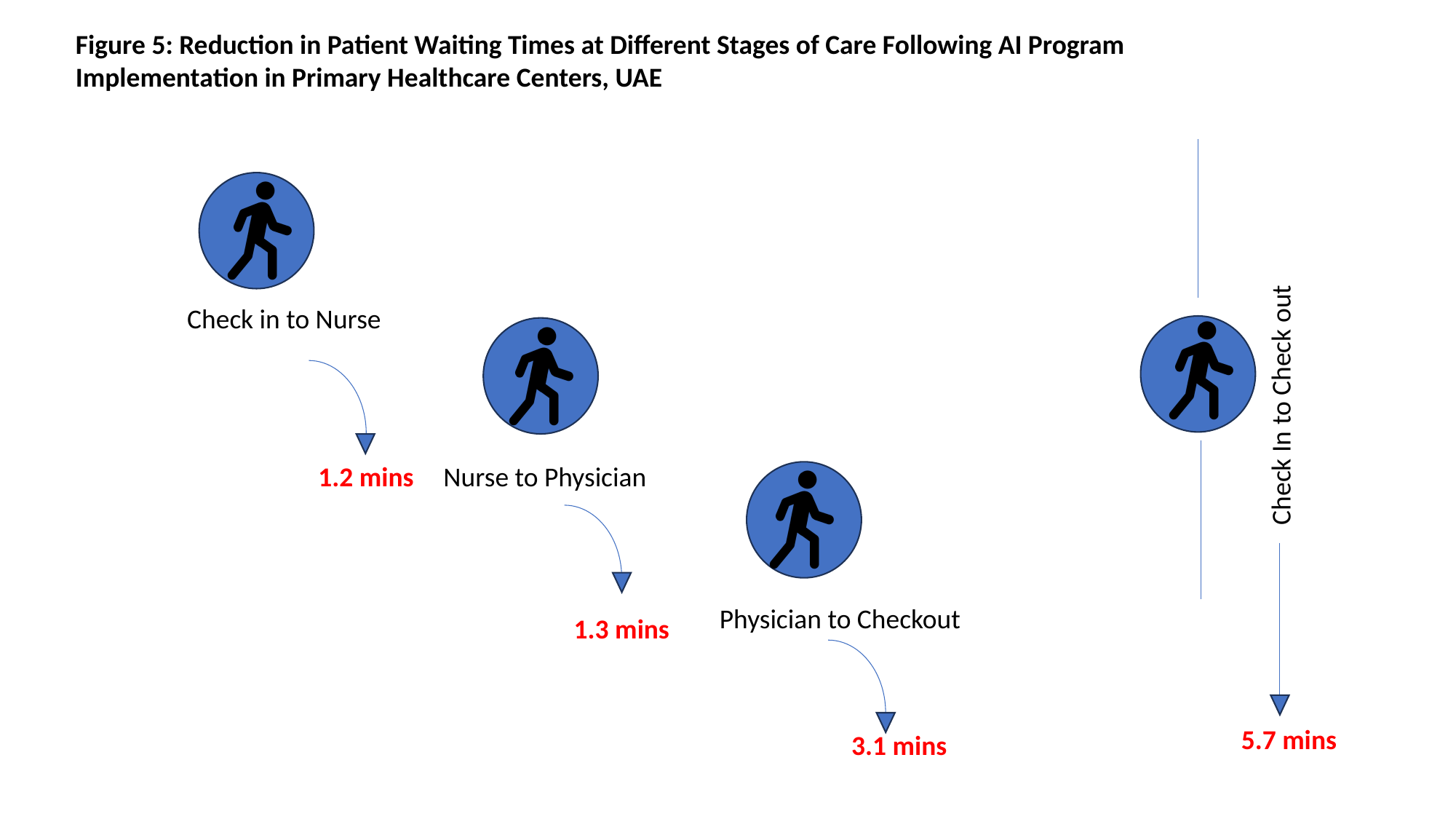

Figure 5: Reduction in Patient Waiting Times at Different Stages of Care Following AI Program Implementation in Primary Healthcare Centers, UAE
Check in to Nurse
Check In to Check out
1.2 mins
Nurse to Physician
Physician to Checkout
1.3 mins
5.7 mins
3.1 mins
